# Mechanical Properties and Morphologies of Carboxyl-Terminated Butadiene Acrylonitrile Liquid Rubber/Epoxy Blends Compatibilized by Pre-Crosslinking

**DOI:** 10.3390/ma9080640

**Published:** 2016-07-29

**Authors:** Shiai Xu, Xiaoxue Song, Yangben Cai

**Affiliations:** 1 School of Chemical Engineering, Qinghai University, Xining 810016, China; 2 School of Materials Science and Engineering, East China University of Science and Technology, Shanghai 200237, China; 030120460@mail.ecust.edu.cn (X.S.); 020130035@mail.ecust.edu.cn (Y.C.)

**Keywords:** epoxy, liquid rubber, mechanical properties, rubber-toughened epoxy, compatibilization

## Abstract

In order to enhance the compatibilization and interfacial adhesion between epoxy and liquid carboxyl-terminated butadiene acrylonitrile (CTBN) rubber, an initiator was introduced into the mixture and heated to initiate the cross-linking reaction of CTBN. After the addition of curing agents, the CTBN/epoxy blends with a localized interpenetrating network structure were prepared. The mechanical properties and morphologies of pre-crosslinked and non-crosslinked CTBN/epoxy blends were investigated. The results show that the tensile strength, elongation at break and impact strength of pre-crosslinked CTBN/epoxy blends are significantly higher than those of non-crosslinked CTBN/epoxy blends, which is primarily due to the enhanced interfacial strength caused by the chemical bond between the two phases and the localized interpenetrating network structure. Both pre-crosslinked and non-crosslinked CTBN/epoxy blends show a bimodal distribution of micron- and nano-sized rubber particles. However, pre-crosslinked CTBN/epoxy blends have smaller micron-sized rubber particles and larger nano-sized rubber particles than non-crosslinked CTBN/epoxy blends. The dynamic mechanical analysis shows that the storage modulus of pre-crosslinked CTBN/epoxy blends is higher than that of non-crosslinked CTBN/epoxy blends. The glass transition temperature of the CTBN phase in pre-crosslinked CTBN/epoxy blends increases slightly compared with the CTBN/epoxy system. The pre-crosslinking of rubber is a promising method for compatibilization and controlling the morphology of rubber-modified epoxy materials.

## 1. Introduction

Epoxy resin is one of the most popular thermosetting materials used as a matrix for adhesives, coatings, sealants and composites because of its excellent mechanical and chemical properties [1,2]. The major drawbacks of epoxy resin are its brittleness and poor resistance to crack propagation. Many methods have been developed to toughen epoxy resins [3,4,5,6,7], among which the incorporation of liquid rubbers, such as butadiene acrylonitrile copolymer [8,9,10,11,12], hydroxyl-terminated polybutadiene (HTPB) [13,14], and natural rubber [15,16], appears to be the most successful approach. It is known that two materials with similar solubility parameters should be compatible with each other. Functionalization can increase the cohesive energy of the functionalized rubber, thus resulting in an increase in the solubility parameters, and consequently the compatibility between epoxy and rubber. The interfacial adhesion can be greatly enhanced by the reaction between the functional groups of liquid rubber and the epoxy or hardener [17]. Carboxyl-terminated butadiene acrylonitrile liquid rubber (CTBN) has been widely used to toughen epoxy resins [8,18,19,20]. Thomas et al. [8] studied the effects of CTBN loadings on the mechanical and thermal properties of cured epoxy blends, and found that the cured epoxy blends containing 15 wt. % of CTBN with an average domain size of 0.5–1.0 μm showed the best balance of properties, and the glass transition temperature (*T*_g_) of the epoxy matrix decreased with the addition of CTBN. Akbari et al. [9] prepared a series of blends by mixing the epoxy resin with 0–25 phr CTBN using dicyandiamide as the curing agent. The results showed that the maximum toughness was achieved at 15 phr CTBN, and the scanning electron microscope (SEM) observation revealed a two-phase morphology of the blends where the rubber particles were dispersed in the epoxy matrix.

Some morphological parameters of the rubber, such as the content, particle size and volume, can strongly influence the mechanical properties of rubber-modified epoxy resins. The toughness of rubber/epoxy blends increased with the increasing rubber content and reached a maximum at 15–20 phr CTBN [9,21]. Large particles were not as effective as small ones in improving the fracture properties of rubber-toughened epoxy resins [22]. In addition, the compatibility and chemical bonds between the rubber and epoxy can also strongly affect the mechanical properties of rubber/epoxy blends. Russell and Chartoff [23] found that the acrylonitrile content of rubber affected both the rubber particle size and volume fraction. Chen and Jan [24] reported that when CTBN was encapsulated with diglycidyl ether of propylene glycol, the fracture energy of CTBN/epoxy could be significantly increased due to the improved interfacial strength. The pre-reaction of CTBN with the epoxy resin in the presence of a catalyst would provide a chemical bond between the dispersed rubber phase and the matrix, leading to a better toughening effect [7,25]. Curing temperature can also greatly affect the morphologies of rubber/epoxy blends as it controls the diffusion of phase-separated rubber and the gelation of the epoxy curing system [26,27].

As the curing reaction proceeds, the molecular weight of the epoxy increases and the rubber phase precipitates, leading to the formation of a two-phase morphology [28]. The morphological development of the rubber/epoxy blends depends largely on the competition between the curing and the phase separation rate, which determines whether the mechanism of the reaction-induced phase separation is nucleation and growth or spinodal de-mixing [29,30]. This is very crucial for the final morphologies and properties of the epoxy/rubber blends. Thus, some special morphologies can be obtained by controlling the curing and phase separation processes. Hsu and Liang [31] prepared novel CTBN/epoxy blends with an interpenetrating network structure and smooth morphology, which were quite different from the conventional CTBN/epoxy blends in that they were capable of suppressing the phase separation process.

In our previous study, we prepared an in situ pre-crosslinked CTBN/epoxy blend with much better mechanical properties compared to its traditional CTBN-modified counterpart. A local interpenetrating structure was formed in the pre-crosslinked CTBN/epoxy blend, which greatly improved the compatibilization and interfacial adhesion between the two phases, and thus resulted in good mechanical properties of the blend [32]. However, the rubber used had a high acrylonitrile content (25%), and thus it was miscible with epoxy. In order to further clarify whether pre-crosslinking helps to improve the miscibility between rubber and epoxy, this study extends our previous study by using CTBN with a much lower acrylonitrile content. Then, the morphologies and mechanical properties of pre-crosslinked CTBN/epoxy blends were compared with those of non-crosslinked CTBN/epoxy blends.

## 2. Experimental

### 2.1. Materials

Diglycidyl ether of bisphenol-A (DGEBA; E51, epoxide equivalent weight = 182–192 g/equivalent) was purchased from Shanghai Resins Co., Ltd. (Shanghai, China). The curing agent isophorone diamine (IPDA) was purchased from Aladin Co., Ltd. (Stockholm, Sweden). Benzoyl peroxide (BPO) was purchased from Shanghai Lingfeng Chemical Reagent Co., Ltd. (Shanghai, China). The liquid rubber used in this study was a CTBN copolymer containing 9% acrylonitrile (CTBN-9) and 0.615 mmol/g of carboxyl, and was purchased from Zibo Qilong Chemical Industry Co., Ltd. (Zibo, China). All materials were used as received, except that BPO was recrystallized from ethanol solution prior to use.

### 2.2. Sample Preparation

Pre-crosslinked or non-crosslinked CTBN/epoxy blends containing 0, 5, 10 or 15 phr CTBN (relative to 100 g epoxy) were prepared in appropriate quantities as described by Zhou et al. [33] and Xu et al. [34]. Then, 24 phr IPDA was added and stirred vigorously to obtain a homogeneous mixture, which was then degassed and poured into preheated iron mould. The curing procedure was 80 °C for 5 h and then heated to 120 °C for 1 h. In this study, X-CTBN(Y)/E51 is used to denote different CTBN/epoxy blends, where X is the CTBN content (phr), and Y is the BPO content (%) proportional to the CTBN content.

### 2.3. Characterization

#### 2.3.1. Fourier-Transform Infrared (FTIR) Spectroscopy

The FTIR spectra of pre-crosslinked and non-crosslinked CTBN/epoxy mixtures without the curing agent were recorded by a Nicolet 6700 spectrometer (Thermal Nicolet, Waltham, MA, USA) operating from 4000 to 500 cm^−1^ at a resolution of 4 cm^−1^.

#### 2.3.2. Mechanical Properties

The tensile tests were performed using a universal testing machine (MTS, Eden Prairie, MN, USA) at a crosshead speed of 2 mm/min according to ASTM D-638. The Izod impact strength was determined using a cantilever impact tester (Chengde Instruments, Chengdu, China) according to ASTM D-256. The average of at least five samples was reported.

#### 2.3.3. Morphology

The tensile fractured and cryofractured surfaces of samples were observed using an S-4800 scanning electron microscope (SEM, Hitachi Ltd., Tokyo, Japan) with an accelerating voltage of 15 kV. The cryofractured surfaces were prepared in liquid nitrogen, and then etched in toluene for 10 h. The surfaces were sputter-coated with gold prior to observation.

#### 2.3.4. Dynamic Mechanical Analysis (DMA)

The dynamic mechanical properties of samples (rectangular bars of 30 × 12 × 3 mm^3^) were determined using a DMA Q800 (TA Instruments, New Castle, DE, USA) at a frequency of 1 Hz and a heating rate of 5 °C/min from −100 to 200 °C.

## 3. Results and Discussion

### 3.1. FTIR Spectra

**Figure 1:**
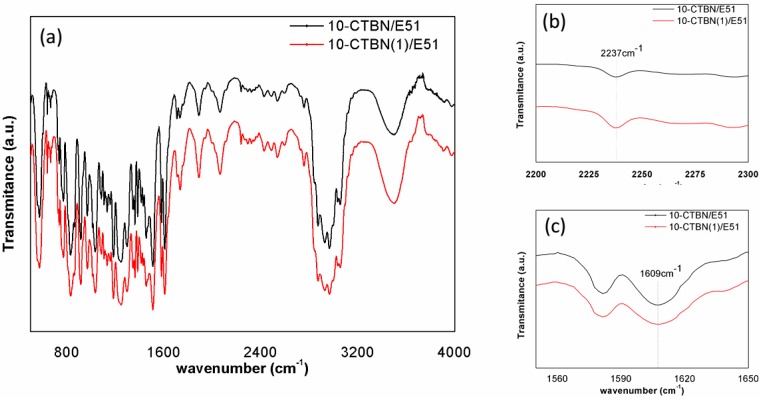
FTIR spectra of pre-crosslinked and non-crosslinked CTBN/epoxy blends: (**a**) full-scale spectra; (**b**) amplified spectra of nitrile groups; and (**c**) amplified spectra of double carbon bonds.

FTIR spectroscopy is able to accurately identify the characteristic groups of CTBN after pre-crosslinking [35]. The FTIR spectra of pre-crosslinked and non-crosslinked CTBN/epoxy blends are shown in Figure 1. It can be seen that the spectral shape of pre-crosslinked CTBN/epoxy blends is almost the same as that of non-crosslinked CTBN/epoxy blends.

In order to quantitatively characterize the variation of double bonds in CTBN, the nitrile group is selected as an internal standard due to the single peak at 2237 cm^−1^. This method is based on the relative areas of the characteristic transmittance peaks of the –C=C– stretching vibration and the –C≡N stretching vibration of CTBN at 1609 and 2237 cm^−1^, respectively. The results are listed in Table 1. It shows that the area ratio of the double carbon bonds to the nitrile groups decreases from 47.79 before pre-crosslinking to 27.07 after pre-crosslinking. Since the nitrile groups remain unchanged during the pre-crosslinking process, the decrease of the area ratio indicates that the polyaddition reaction between the double carbon groups is initiated by BPO via the free radical mechanism [31].

### 3.2. Mechanical Properties

The tensile strength, tensile modulus, elongation at break and impact strength of different CTBN/epoxy blends are given in Table 2. As the concentration of CTBN increases from 5 to 15 phr, the tensile strength and modulus decrease because the tensile strength and modulus of rubber are much lower than that of the epoxy matrix [8,36]. Besides, the elongation at break increases lightly, and the impact strength increases from 1.25 to 1.78 kJ/m^2^. Several toughening mechanisms have been proposed for CTBN-toughened epoxies [37]. The cavitation process and crack pinning of rubber particles have been shown to play key roles [32,34]. Increasing the CTBN concentration leads to an increase in the number of CTBN particles, and thus an increase in the impact strength.

More importantly, the mechanical properties of pre-crosslinked CTBN/epoxy blends are much better than those of non-crosslinked CTBN/epoxy blends. Pre-crosslinked CTBN/epoxy blends show a higher modulus than non-crosslinked CTBN/epoxy blends. The tensile strength, elongation at break and impact strength of 5 phr pre-crosslinked CTBN/epoxy blends are increased by 19.9%, 30.2% and 30.7% as compared with those of 5 phr non-crosslinked CTBN/epoxy blends, indicating that pre-crosslinked CTBN is better than the non-crosslinked one in modifying epoxy resin. It is also noted that the mechanical properties of pre-crosslinked CTBN/epoxy blends increase with the increase of the BPO content and reach an optimum at 1.5% BPO, after which a further increase in the BPO content results in a slight decrease in the mechanical properties. Hereafter, the BPO content is 1.5% in all crosslinked CTBN/epoxy blends. Hsu and Liang [31] also reported that the toughness of CTBN/epoxy blends had a similar trend with the increase of the BPO content.

Although rubber does not participate in the curing reaction, but its molecular weight has a significant effect on its miscibility with epoxy monomer and the kinetics of phase separation in curing, which finally influences the phase separation of the curing system. Therefore, the final morphology of the blends can be controlled by adjusting the molecular weight of the rubber.

In the preparation of non-crosslinked CTBN/epoxy blends by the traditional method, CTBN was first dissolved in epoxy, and began to precipitate after the addition of the curing agent to form a second phase as the molecular weight of the epoxy increased. CTBN molecules tended to migrate and agglomerate into large particles during phase separation because of the low molecular weight of CTBN. Theoretically, the carboxyl groups in CTBN could react with the epoxide groups of the epoxy and the amino groups of the curing agent. However, only a small portion of CTBN molecules could react with the matrix because the reaction rate of the carboxyl groups was low due to the competing reaction between epoxides and the amino groups [34]. The weak chemical linkages between the rubber phase and the matrix did not allow for the effective transfer of stress under external forces, thus leading to a decrease of yield strength and Young’s modulus after the addition of CTBN. In the pre-crosslinking process, the homogeneous mixture of epoxy resin with CTBN was heated in the presence of an initiator. The initiator could break the double bond of C=C in liquid rubber, which initiated the crosslinking of CTBN molecules with each other, and thus resulted in an increase of the CTBN’s molecular weight [32,33]. CTBN molecules with a high molecular weight migrated slowly in the phase separation. Moreover, the carboxyl groups in CTBN could fully react with epoxy during the pre-crosslinking to generate copolymers in the absence of the curing agent. Thus, more chemical linkages were formed between CTBN and the matrix. These copolymers aggregated on the interface between the two phases, which also hindered the migration of the CTBN and epoxy molecules between the two phases in the phase separation. Thus, a portion of the epoxy was wrapped in the dispersed crosslinked rubber phase. As the curing proceeded, a local interpenetrating polymer network was formed in the dispersed rubber phase [32], which contributed greatly to improve the compatiblization and interfacial adhesion between the two phases, and therefore resulted in much better mechanical properties of pre-crosslinked CTBN/epoxy blends.

### 3.3. Morphology

**Figure 2:**
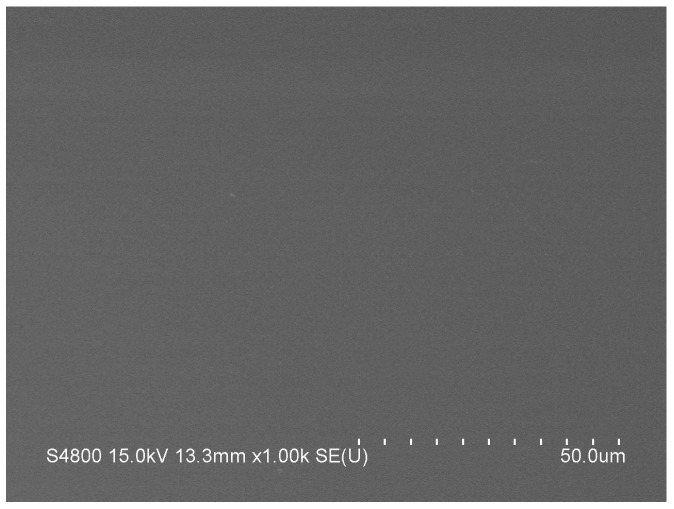
SEM micrograph of the tensile fractured surfaces of neat epoxy.

**Figure 3:**
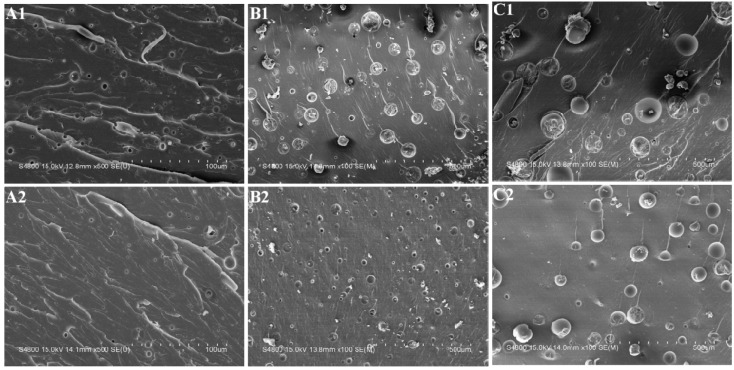
SEM micrographs of the tensile fractured surfaces: (**A1**) 5 phr CTBN/epoxy blends; (**A2**) 5 phr pre-crosslinked CTBN/epoxy blends; (**B1**) 10 phr CTBN/epoxy blends; (**B2**) 10 phr pre-crosslinked CTBN/epoxy blends; (**C1**) 15 phr CTBN/epoxy blends; and (**C2**) 15 phr pre-crosslinked CTBN/epoxy blends at a low magnification.

The tensile fractured surfaces of neat epoxy, pre-crosslinked and non-crosslinked CTBN/epoxy blends were observed by SEM. Figure 2 shows that the fractured surface of neat epoxy is mirror-like, which is characteristic of the brittle materials. However, the pre-crosslinked and non-crosslinked CTBN/epoxy blends are quite rough, indicating massive shear deformation (Figure 3). A large number of stress whitening zones are formed in CTBN-modified epoxy resins due to the scattering of visible light from the layer of scattering centers, which are voids caused by the cavitation of rubber particles [37,38,39]. The stress whitening dissipates a large amount of energy, which explains why CTBN-modified epoxy exhibits a higher impact strength than the neat epoxy.

The fractured surfaces of all CTBN/epoxy blends show a two-phase morphology with a continuous epoxy phase and a dispersed rubber phase of spherical particles. CTBN is initially miscible with epoxy, but will undergo phase separation during the curing reaction, which is called “reaction-induced phase separation”. As the CTBN concentration increases, the rubber domain size increases. This is in agreement with other CTBN/epoxy blends [20,40], and it is associated with the reagglomeration and coalescence of dispersed rubber particles [41].

**Figure 4:**
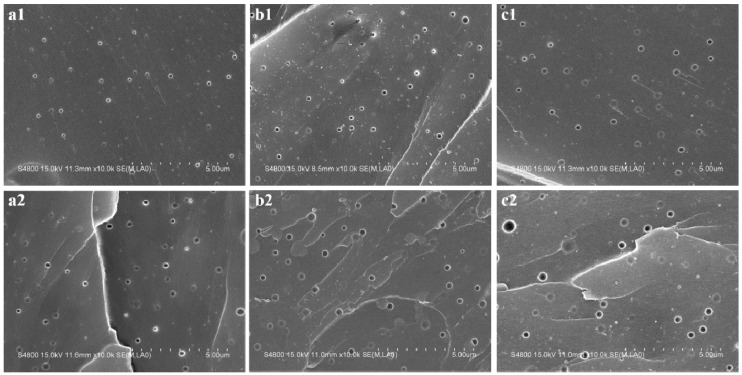
SEM micrographs of cryofractured surfaces etched by toluene: (**a1**) 5 phr CTBN/epoxy blends; (**a2**) 5 phr pre-crosslinked CTBN/epoxy blends; (**b1**) 10 phr CTBN/epoxy blends; (**b2**) 10 phr pre-crosslinked CTBN/epoxy blends; (**c1**) 15 phr CTBN/epoxy blends; and (**c2**) 15 phr pre-crosslinked CTBN/epoxy blends at a high magnification.

In order to compare the size and distribution of rubber particles in two different systems, the samples were fractured in liquid nitrogen, then etched by toluene, and the cryofractured surfaces of the blends are shown in Figure 4. Both pre-crosslinked and non-crosslinked CTBN/epoxy blends show a bimodal distribution of the rubber particle sizes (micron- and nano-sized) due to the heterogeneity of the molecular weight and the acrylonitrile content in the CTBN chains. A higher acrylonitrile content in the CTBN chains leads to a better solubility in epoxy, thus requiring a higher degree of curing before the phase separation of CTBN. The viscosity of the epoxy phase is also higher at a higher curing degree, which could reduce the ease of CTBN diffusion and of particle agglomeration, and thus results in smaller particle sizes [42]. Most CTBN molecules have a high molecular weight and a low acrylonitrile content, thus resulting in poor miscibility with the epoxy matrix and large phase-separated rubber particles, while a small number of CTBN molecules with a low molecular weight and a high acrylonitrile content are dispersed in the matrix in nano-size.

The uniform distribution of the rubber particles in the matrix allows the plastic shear yielding to operate through the matrix [43]. The deformation lines are propagated through the rubber domains, promoting stress transfer between the particles and the epoxy matrix. Such plastic deformation occurs to a greater extent in pre-crosslinked CTBN/epoxy blends due to the enhanced interactions between the two phases, leading to increased energy dissipation and toughness. The crack pinning theory suggests that the interaction of a propagating crack front with the obstacles (rubber particles) results in a higher toughness [44,45]. However, the propagating crack may be pinned by the particles so that the crack front between the particles moves on and extends by bowing, which leads to enhanced line energy and crack resistance [46]. Figure 4 shows the bowing of the crack front between the rubber particles of the pre-crosslinked CTBN/epoxy blends.

The number-average diameter (*D*_n_) and weight-average diameter (*D*_w_) of the dispersed rubber particles in different rubber-modified epoxies were calculated using the following equations:

(1) $$D_{i}=\sum n_{i}D_{i}/n_{i}$$

(2) $$D_{w}=\sum n_{i}D_{i}^{2}/n_{i}D_{i}$$

(3) $$PDI=D_{w}/D_{n}$$

where *n*_i_ is the number of particles in the diameter range *D*_i_, and *PDI* is the polydispersity index.

Table 3 shows that the *D*_n_ and D_w_ values of both micron-sized and nano-sized particles increase with the increasing rubber content. It is noted that the *D*_n_ and *D*_w_ values of micron-sized rubber particles are smaller in the pre-crosslinked CTBN/epoxy blends than in the non-crosslinked CTBN/epoxy blends with the same rubber content, while the opposite is observed for nano-sized rubber particles. The morphological development of rubber/epoxy blends depends on not only the thermodynamics, but also the kinetics of the phase separation, and thus the competition between the curing and the phase separation determines the mechanism of the reaction-induced phase separation, which is crucial for the final morphologies and properties of the epoxy/rubber blends [17]. However, if the curing reaction proceeds quickly, the viscosity of the system increases sharply and the phase separation is frozen before reaching equilibrium. As a result, the composition of the blend may differ greatly from that at equilibrium, resulting in an inhomogeneous particle sizes of CTBN. For the pre-crosslinked system, the molecular weight of rubber is high, and thus the phase separation is initiated earlier in curing [10]. At this time, the viscosity of the system is still low, the phase separation proceeds easily, and the composition of the blends is similar to that at the thermodynamic equilibrium, resulting in homogeneous CTBN particle sizes. This may explain why the pre-crosslinked CTBN/epoxy blends have larger nano-sized rubber particles and smaller micron-sized rubber particles than the CTBN/epoxy blends.

### 3.4. Dynamic Mechanical Properties

**Figure 5:**
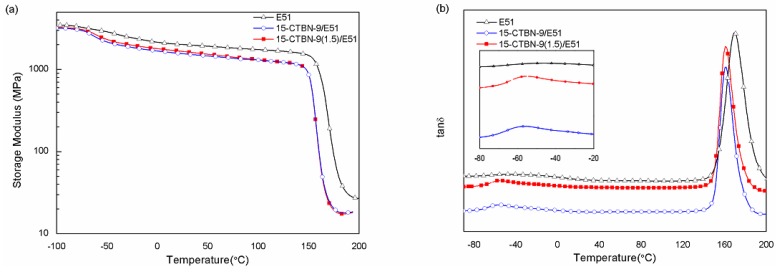
Variations of (**a**) storage modulus and (**b**) tan δ with temperature for different CTBN/epoxy blends.

The dynamic mechanical properties of neat epoxy and different CTBN/epoxy blends are shown in Figure 5. The storage modulus of all samples decreases with the increasing temperature, and that of epoxy decreases after the addition of CTBN (Figure 5a). This is due to the presence of low-modulus rubber particles and the improved flexibility of CTBN/epoxy blends [25,47]. The storage modulus of pre-crosslinked CTBN/epoxy blends is higher than that of non-crosslinked CTBN/epoxy blends due to the enhanced interfacial strength between the rubber and epoxy phases [32]. However, their moduli are almost the same at higher temperatures (>100 °C), because the systems are viscous at high temperatures, and the interfacial adhesion is not a key factor for stress transfer.

Figure 5b shows the variation of tan δ with temperature for neat epoxy and different CTBN/epoxy blends. For neat epoxy, the broad and flat peak at low temperatures is identified as the β relaxation of epoxy, which is often attributed to the crankshaft motions of glycidyl groups [36,46,48]; further, the relaxation peak at high temperatures is identified as the α relaxation of epoxy, which corresponds to the glass transition of epoxy [48]. As expected, the α relaxation peak of the epoxy phase shifts to a lower temperature in rubber-modified epoxy, because CTBN rubber has a much lower glass transition temperature than the epoxy matrix. When the temperature exceeds the glass transition zone of CTBN, the CTBN phase is in a high-elastic state, which could improve the chain mobility of epoxy and thus result in the depression of the glass transition temperature of the epoxy matrix. A small amount of rubber with a high nitrile content and a low molecular weight dissolves in the epoxy and plasticizes the epoxy, resulting in a slight decrease of the glass transition temperature of the epoxy matrix [49]. Two distinct relaxation peaks are observed in the tan δ curves for both CTBN/epoxy blends (at ca. 165 °C and −55 °C for the glass transition of epoxy and CTBN, respectively) [50]. The addition of 15 phr pre-crosslinked CTBN does not alter the α peak of the epoxy phase, while the relaxation peak of rubber shifts slightly from −56.4 °C to −54.7 °C (enlarged view) as compared with that of 15 phr non-crosslinked CTBN/epoxy blends. This is due to the decreased mobility of CTBN as a result of crosslinking. Besides, some embedded epoxy in the rubber domains in pre-crosslinked CTBN/epoxy blends also exists, which could be responsible for the increased glass transition temperature of pre-crosslinked CTBN [51]. Although the glass transition region of CTBN partly overlaps with the β relaxation of epoxy, they can be distinguished from each other as the CTBN relaxation peak is sharper and its intensity is proportional to the rubber content [41].

## 4. Conclusions

Pre-crosslinked and non-crosslinked CTBN/epoxy blends containing 0, 5, 10 and 15 phr CTBN were prepared, and their mechanical properties were compared in this study. The FTIR results show that free radical polymerization reactions take place between the double carbon bonds of CTBN during the pre-crosslinking. The tensile properties and impact strength of pre-crosslinked CTBN/epoxy blends are significantly higher than those of non-crosslinked CTBN/epoxy blends; which is mainly due to the enhanced interfacial strength caused by the localized interpenetrating network. The mechanical properties of pre-crosslinked CTBN/epoxy blends increase with the increasing BPO content and reach an optimum at 1.5% BPO, after which a further increase in the BPO content results in a slight decrease in the mechanical properties. Both pre-crosslinked and non-crosslinked CTBN/epoxy blends show a bimodal distribution of rubber particle sizes (micron- and nano-sized). However, pre-crosslinked CTBN/epoxy blends have smaller micron-sized rubber particles and larger nano-sized rubber particles than non-crosslinked CTBN/epoxy blends. There is a slight increase in the storage modulus and glass transition temperature of the rubber phase after the pre-crosslinking. The use of pre-crosslinked rubber is a promising method for controlling the morphology of rubber-modified epoxy materials.

## Figures and Tables

**Table 1:** Peak area and area ratio of the double carbon bonds to the nitrile groups in CTBN/epoxy blends.

Sample	Area-_C_^a^	Area-_N_^a^	Area Ratio
10-CTBN/E51	9.08	0.19	47.79
10-CTBN(1.5)/E51	8.39	0.31	27.07

^a^ Area-_C_ and Area-_N_ mean the peak area of the double carbon bonds and the nitrile groups in the FTIR spectra, respectively.

**Table 2:** Mechanical properties of neat epoxy, pre-crosslinked and non-crosslinked CTBN/epoxy blends.

Sample	Modulus (MPa)	Tensile Strength (MPa)	Strain at Break (%)	Impact Strength (kJ/m^2^)
Neat epoxy	2917 ± 16	78.7 ± 2.4	4.2 ± 0.7	1.25 ± 0.14
5-CTBN/E51	2601 ± 26	64.9 ± 1.9	4.3 ± 0.3	1.40 ± 0.17
5-CTBN(0.5)/E51	2745 ± 19	71.6 ± 2.6	4.5 ± 0.3	1.73 ± 0.06
5-CTBN(1.0)/E51	2754 ± 30	73.5 ± 1.3	5.0 ± 0.3	1.78 ± 0.23
5-CTBN(1.5)/E51	2773 ± 25	77.8 ± 0.8	5.6 ± 0.5	1.83 ± 0.11
5-CTBN(2.0)/E51	2695 ± 17	71.4 ± 1.8	4.6 ± 0.4	1.70 ± 0.23
10-CTBN/E51	2401 ± 32	61.1 ± 1.6	4.5 ± 0.3	1.68 ± 0.06
10-CTBN(0.5)/E51	2492 ± 42	66.6 ± 1.0	4.9 ± 0.4	1.71 ± 0.28
10-CTBN(1.0)/E51	2469 ± 12	67.6 ± 0.4	5.3 ± 0.3	1.81 ± 0.16
10-CTBN(1.5)/E51	2476 ± 22	68.0 ± 0.5	5.8 ± 0.5	1.90 ± 0.30
10-CTBN(2.0)/E51	2467 ± 17	63.8 ± 0.7	5.2 ± 0.1	1.74 ± 0.12
15-CTBN/E51	2135 ± 28	53.8 ± 0.9	4.7 ± 0.2	1.78 ± 0.20
15-CTBN(0.5)/E51	2244 ± 23	58.8 ± 0.5	5.0 ± 0.8	1.83 ± 0.06
15-CTBN(1.0)/E51	2235 ± 30	58.9 ± 0.4	5.4 ± 0.8	1.93 ± 0.08
15-CTBN(1.5)/E51	2253 ± 15	60.0 ± 0.3	6.6 ± 0.7	1.95 ± 0.14
15-CTBN(2.0)/E51	2191 ± 7	57.6 ± 0.7	5.0 ± 0.3	1.80 ± 0.12

**Table 3:** Dispersed particle sizes and polydispersity of different CTBN/epoxy blends.

Samples	Micron-Sized Particles			Nano-Sized Particles		
	*D*_n_ (μm)	*D*_w_ (μm)	PDI	*D*_n_ (nm)	*D*_w_ (nm)	PDI
5-CTBN/E51	4.18	5.74	1.37	134	143	1.07
5-CTBN(1.5)/E51	2.71	4.48	1.65	201	227	1.13
10-CTBN/E51	51.12	58.13	1.14	188	198	1.05
10-CTBN(1.5)/E51	25.00	29.26	1.17	271	291	1.07
15-CTBN/E51	75.14	91.75	1.22	190	209	1.10
15-CTBN(1.5)/E51	57.00	67.54	1.18	276	310	1.12
